# Viability-PCR Shows That NAAT Detects a High Proportion of DNA from Non-Viable *Chlamydia trachomatis*

**DOI:** 10.1371/journal.pone.0165920

**Published:** 2016-11-03

**Authors:** Kevin J. H. Janssen, Christian J. P. A. Hoebe, Nicole H. T. M. Dukers-Muijrers, Lisanne Eppings, Mayk Lucchesi, Petra F. G. Wolffs

**Affiliations:** 1 Department of Medical Microbiology, School of Public Health and Primary Care (CAPHRI), Maastricht University Medical Center (MUMC+), Maastricht, The Netherlands; 2 Department of Sexual Health, Infectious Diseases and Environmental Health, South Limburg Public Health Service (GGD South Limburg), Geleen, The Netherlands; University of Texas Health Science Center at San Antonio, UNITED STATES

## Abstract

**Objectives:**

According to the current guidelines for laboratory diagnosis of sexually transmitted infections (STIs), nucleic acid amplification tests (NAATs) are the preferred diagnostic method for *Chlamydia trachomatis* (CT) infections. However, NAATs amplify the available target DNA without discriminating between DNA originating from viable or non-viable CT. Assessing CT viability will provide more insights in the clinical and public health relevance of a CT positive test result. The aim of this study was to technically validate and implement viability-PCR (V-PCR) to asses CT viability.

**Methods:**

Technical validation of V-PCR was performed by the assessment of predefined viability ratios of CT. Samples were subjected to V-PCR which consisted of propidium monoazide (PMA) treatment prior to DNA extraction followed by quantitative PCR (qPCR) targeting the *ompA* gene for the detection of CT DNA. Finally, V-PCR was applied to vaginal swabs of 50 CT positive patients, as indicated by routine NAAT, collected at our outpatient STD clinics before antimicrobial treatment.

**Results:**

Technical validation of V-PCR showed that PMA treatment of heat-inactivated CT culture resulted in an almost complete loss of qPCR signal. PMA treated samples of the fresh viable CT culture showed no marked reduction of PCR signal, indicating that all DNA from viable CT could be detected. Applying V-PCR to clinical samples showed that in 36% of samples (18/50) less than 1% of CT DNA originated from viable bacteria.

**Conclusions:**

V-PCR showed to be a fast and easy method to assess CT viability in clinical samples, without the need of traditional challenging cell culture methods. Furthermore, V-PCR results of clinical samples have indicated that a substantial amount of the amplified CT DNA originated from non-viable cells. Although results might be influenced by cell death during transport, this study suggests that there is a potential overestimation of quantitative CT positivity by currently used NAATs.

## Introduction

*Chlamydia trachomatis* (CT) infections are the most commonly reported bacterial sexual transmittable infections (STIs) in the world. The World Health Organization (WHO) estimated an incidence of 131 million new CT cases in women and men aged 15–49 years globally in 2012 [1]. Current guidelines for laboratory diagnosis of STIs strongly recommend nucleic acid amplification tests (NAATs) for the detection of CT nucleic acid (DNA or RNA) in clinical samples [2–4], due to their high sensitivity (>90%), specificity (≥99%) and short turnaround times [5–7]. However, NAATs have shortcomings, recently reviewed by Trembizki *et al*. [8], to be kept in mind when interpreting test results. One of the main disadvantages of NAAT assays is that the available target DNA is amplified without discriminating between DNA originating from viable or non-viable CT. Subsequently, DNA amplification of non-viable CT potentially results in an overestimation of quantitative test positivity by currently used NAATs. Several recent studies have shown that a CT positive test result could be detected up to 8 weeks even after state-of-the-art treatment [9–11]. Until today, the clinical implications (e.g. sequelae, treatment and screening management) and public health implications (e.g. transmission) of prolonged CT test positivity are still unclear [11, 12]. The assessment of CT viability is essential to gain more insight in these impacts.

Traditionally, cell culture is the gold standard as it approaches a specificity of 100% for the assessment of viability. However, cell culture methods are labour-intensive, technically demanding, expensive, and lack sensitivity for the detection of CT infections (range from 70–85%) [13]. One promising strategy to assess microbial viability relies on the use of membrane impermeable DNA intercalating dyes as a sample pre-treatment before conducting molecular techniques, also called viability-PCR (V-PCR). This approach has been first described by Nogva et al. in 2003 and was further improved by Nocker et al. in 2006 by using propidium monoazide (PMA) instead of ethidium monoazide (EMA) [14–16]. In short, samples are split into two aliquots and one of the aliquots is treated with PMA. The remaining aliquot is left untreated. After a short incubation period, PMA treated samples are exposed to a light source, which catalyses the irreversible formation of crosslinks between PMA and any accessible DNA molecule (e.g. DNA from membrane impaired bacteria). The crosslinking between PMA and DNA makes the target DNA unavailable for detection by quantitative PCR (qPCR). Subsequently, both aliquots are subjected to DNA extraction and qPCR analysis. The resulting difference in qPCR signal between the PMA treated and untreated aliquot corresponds with the amount of target DNA in the sample originating from non-viable bacteria [15].

To our knowledge, V-PCR has never been applied in the field of bacterial STI diagnostics. Moreover, V-PCR has only rarely been applied for the assessment of intracellular pathogen viability. The objective of our study was to implement and evaluate V-PCR as a new approach to assess CT viability. Therefore we performed a microbiological technical validation and a clinical validation with application on samples from our STI clinic.

## Materials and Methods

### Cell culture and infection with CT

HeLa229 cells were obtained from the American Type Culture Collection (ATCC^®^ CLL-2^™^). Cells were cultured in Minimum Essential Medium (MEM; Life Technologies, Bleiswijk, The Netherlands) supplemented with 10% fetal bovine serum (FBS; Lonza Bio Science, Vierviers, Belgium), 2mM L-glutamine, 1mM sodium pyruvate, and 1% non-essential amino acids (Life Technologies, Bleiswijk, The Netherlands) at 37°C in air containing 5% CO_2_.

CT serotype D (DSM19411; Deutsche Sammlung von Mikroorganismen und Zellkulturen, Braunschweig, Germany) was propagated in HeLa cells according to manufacturer’s protocol. Shortly, CT stock solution was added to a monolayer of HeLa cells in the presence of supplemented MEM medium containing 1 μg/ml cycloheximide (Sigma Aldrich). Infection was completed by centrifugation-assisted inoculation at 900 rcf for 1h at 34°C, and infected cells were incubated at 37°C in air containing 5% CO_2_. After 48 hours, infected cells were lysed by freezing at -80°C to release CT elementary bodies (EBs). Cell debris was pelleted at 200 rcf for 10 minutes at 4°C followed by centrifugation of the EB containing supernatant 30000 rcf for 30 min. The bacterial pellet was washed twice and stored in sucrose-phosphate-glutamate (SPG) buffer (250 mM sucrose, 10 nM sodium phosphate, and 5 mM L-glutamic acid (pH 7.2)) at -80°C for further use.

### DNA extraction

Total nucleic acids from 200 μl sample were isolated using the QIAamp DNA Mini Kit (Qiagen, Hilden, Germany) as per manufacturer’s protocol, and eluted in 120 μl elution buffer. The eluate was stored at -20°C and thawed once for quantification.

### CT quantification

CT qPCR was conducted by the use of primers targeting the single-copy *ompA* gene, coding for the major outer membrane protein (MOMP), as described by Jalal et. al. [17]. QPCR was performed on a 7900HT Real-Time PCR System (Applied Biosystems, Foster City, California), in a total volume of 25 μl per reaction, consisting of 10 μl purified DNA and 15 μl reaction mixture. The qPCR reaction mixture contained 12.5 μl Absolute qPCR Rox Mastermix (Thermo Scientific, Waltham, USA) and 2.5 μl primer/probe mix consisting of 840 nM forward and reverse primer and 100 nM probe. The amplification conditions were 15 minutes of initial activation at 95°C, followed by 42 cycles of 95°C for 15 seconds and 60°C for 60 seconds. Cycle threshold-values were entered into a master calibration curve to determine chlamydial load in log CT/ml, as described elsewhere [18].

### V-PCR

Prior to DNA purification, samples were split in two aliquots and treated with the membrane impermeable DNA binding dye PMA (Biotium, Inc., Hayward, California). PMA was dissolved in 98 μl dH_2_O to obtain a stock concentration of 20 mM and stored at 4°C in the dark. PMA stock solution was added to 500 μl sample to a final concentration of 50 μM. Following an incubation period of 10 min in the dark at 4°C, samples were fixed on a rotor and exposed to a 650-W light source for 10 min. After 5 min of light exposure, samples were placed on ice for 2 min to avoid excessive heating. Total DNA was extracted and stored at -20°C. The optimal treatment conditions and adequate PMA concentration were confirmed and were in line with previous studies (data not shown) [15, 19]. Differences between PMA treated and untreated samples were reported as Δlog CT/ml. Larger values of Δlog CT/ml are associated with a higher amount of target DNA originating from non-viable bacteria.

### Technical validation of V-PCR

To investigate the potential of V-PCR to distinguish between viable and non-viable CT, heat-killed CT culture was mixed with the untreated CT culture in defined ratios with untreated CT culture representing 0%, 0.1%, 1%, 10%, 50% and 100% of the total, respectively. Heat-killing of CT was achieved by incubation of the CT culture at 95°C for 15 min at 200 rpm on an Eppendorf Thermo mixer 5436 (Eppendorf, Hamburg, Germany). Inoculation of heat-killed CT culture onto monolayers of HeLa cells resulted in a complete loss of cultivability (data not shown).

### Clinical sample collection

Genital CT positive women, according to the COBAS 4800 CT/NG NAAT assay (Roche Diagnostics, Basel, Switzerland), were included in this study before anti-biotic treatment. Participants were recruited, after written informed consent, upon return for treatment at the local STI clinics (GGD South-Limburg). All samples were self-collected vaginal swabs and each participant provided two swabs. The first swab was collected and placed in 2SP transport buffer to allow CT viability testing the second swab was stored in COBAS buffer to test for CT positivity by routine NAAT assay.

### Statistical analysis

The descriptive statistics of samples (i.e. median, 25% and 75% percentiles, minimum, and the maximum of CT load) were calculated using GraphPad Prism version 5.03 for Windows (GraphPad Software, San Diego, California, USA, www.graphpad.com). Error bars in figures represent standard deviations from three independent experiments.

## Results

### Effect of PMA on defined viability ratios of CT serotype D

**Fig 1:**
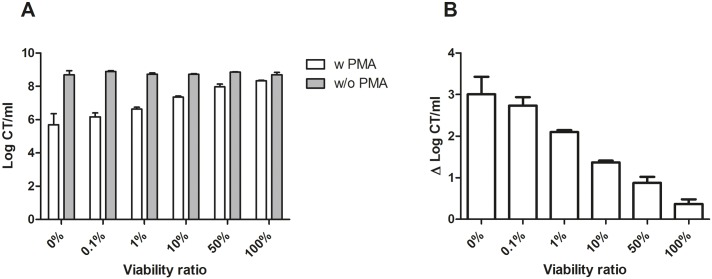
Effect of PMA treatment on defined viability ratios of CT. representing a untreated viable CT culture proportion of 0%, 0.1%, 1%, 10%, 50%, and 100%, respectively. qPCR was performed using primers specific for the chlamydial single-copy *ompA* gene. Error bars represent standard deviations from three independent replicates. (A) Log CT/ml obtained for each viability ratio treated with (w PMA; white bars) and without (w/o PMA; grey bars) PMA. (B) Bars present the change in log CT/ml observed as a result of treatment with PMA prior to DNA purification.

Independent triplicate samples of viability ratios were subjected to V-PCR analysis. Whereas the obtained log CT/ml from viability ratios 0% through 100% were comparable without PMA treatment, increasing proportions of fresh CT culture led to a substantial increase in the acquired log CT/ml after exposure to PMA (Fig 1A). The highest Δlog CT/ml was observed for the sample containing only heat-killed CT culture, i.e. a Δlog CT/ml of 3.01 (±0.42) log units. PMA-treatment of the sample containing only fresh CT culture resulted in a Δlog CT/ml of 0.37 (±0.11) log units. Thus, treatment with PMA of the heat-killed CT culture almost completely inhibited amplification of the target DNA (i.e. a relative qPCR signal reduction of 99.9%), while no marked difference could be detected for the samples of the fresh untreated CT culture. Furthermore, the resulting Δlog CT/ml values decreased gradually with increasing proportions of fresh CT culture for the viability ratios of 0% up to 100% (Fig 1B).

### Application of the V-PCR method to clinical samples

**Fig 2:**
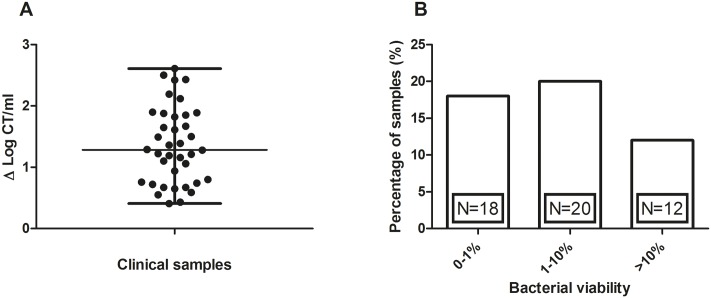
Evaluation of clinical samples by viability-PCR. (A) Δlog CT/ml of the clinical samples are presented as scatter dot plots displaying the median with the interquartile ranges. (B) Clinical samples categorized according to the amount of DNA originating from viable CT.

To further assess the performance of the V-PCR method we collected in our STI clinic a convenience sample of 50 genital CT positive (by NAAT) women before antibiotic treatment. All these clinical samples were self-collected vaginal swabs. The overall median age of 50 participants was 22 years (inter-quartile range (IQR) 21–24). The median transport time of samples, from collection to arrival at the laboratory, was 7.8 hours (IQR 4.3–24.9). In 24% (12/50) of the samples PMA treatment resulted in a total loss of qPCR signal, indicating that the initial detected amount of DNA originated from non-viable CT only. For the remaining samples, V-PCR results ranged from 0.41 to 2.61 Δ log CT/ml with a median of 1.29 Δ log CT/ml (Fig 2A). The application of V-PCR on clinical samples has shown that in 36% (18/50) of samples less than 1% of CT DNA originated from viable CT. Furthermore, in 40% (20/50) of samples between 1% and 10% and only in 24% (12/50) of samples more than 10% of the CT DNA originated from viable bacteria (Fig 2B).

## Discussion

Our study is the first to technically validate V-PCR as a method to assess CT viability and to implement this method in clinical samples. Moreover, to our knowledge, this is the first time V-PCR has been applied in the field of STIs. This study shows that the assessment of CT viability in clinical samples can be achieved by the application of V-PCR, without the need of traditional challenging cell culture methods, as these are labour intensive, technically demanding and lack sensitivity for the diagnosis of CT [20].

In this study, technical validation of the V-PCR method was conducted by the assessment of predefined viability ratios of CT cultures. Results showed that as expected, for all pre-mixed ratios the values without PMA treatment were similar. When applying PMA treatment and comparing to the non-PMA treated values, results have shown that there was only a slight difference in qPCR signal of Δ0.37 (±0.11) log CT/ml for the sample containing fresh CT culture, most likely caused by the presence of non-viable CT in the cultures due to prolonged incubation times (48 hours) rather than PMA membrane leakage. Additionally, this minor difference in qPCR signal between the PMA treated and untreated samples of the fresh culture demonstrates that it is unlikely that PMA bound to non-chlamydial DNA interferes with the CT specific PCR. Furthermore, PMA treatment of samples containing only heat-killed CT was effective in substantially reducing qPCR detection of the target DNA (i.e. qPCR signal reduction of 99.9%). Although Fig 1A shows a remaining 6 log CT/ml after treatment with PMA of the heat-killed CT culture, qPCR signal was reduced by approximately 3 log CT/ml compared to the corresponding sample without PMA treatment. These results are in line with the successful application of V-PCR to distinguish between viable and non-viable organisms in different fields of research such as *Salmonella typhimurium* in food samples [21], *Staphylococcus spp*. commonly involved in prosthetic infections [19], and *Enterococcus faecalis* and *Bacteroides thetaiotaomicron* in waste water samples [22], which demonstrated that PMA treatment of heat-inactivated bacterial cultures resulted in up to a 3–4 log reduction of detectable target sequences compared to the corresponding samples that were not exposed to PMA.

To demonstrate clinical applicability, we have conducted the V-PCR method on self-collected vaginal swab samples from 50 CT positive women before treatment with antibiotics. Resulting data were categorized according to the amount of CT DNA originating from viable cells in the original sample. Interestingly, results have shown that in a remarkable 76% (38/50) of samples less than 10% of CT DNA originated from viable CT, suggesting an overestimation of quantitative CT positivity by routine NAAT assays due to the detection of DNA originating from non-viable CT. Several previous studies already established that PCR detects DNA from non-viable CT [9, 23, 24]. The present study however evaluates the extent of CT viability prior to treatment. Results in this study have shown that in 36% of vaginal swab samples less than 1% of CT DNA originated from viable CT, i.e. more than 2 log CT load decrease of the PMA treated sample compared to the corresponding untreated sample. This decrease in viability is possibly caused by the process of natural clearance of CT infection at the time patients returned for treatment. In line with our results, natural clearance of CT DNA and/or RNA during the period between CT detection and treatment has previously been assessed and varied between 9–44% in urogenital samples [25, 26]. Furthermore, in a recent study by our research group we have reported that CT load during the period between CT detection and treatment decreased more than 90% in one out of five of vaginal swab samples [27].

Our study has limitations. V-PCR correlates viability solely based on bacterial cell membrane integrity [15]. Thus, V-PCR may overestimate the amount of viable bacteria in the presence of non-viable bacteria of which membrane integrity has not been affected [28]. Furthermore, results of tested clinical samples may have been influenced by bacterial cell death during transport, which could result in an overestimation of the amount of non-viable CT DNA present in the fresh collected samples. Therefore, the time between sample collection and laboratory processing for V-PCR should be as short as possible to achieve the most accurate estimation of CT viability. Moreover, consecutive sampling could potentially slightly influence test results. Therefore, we used the first sample for V-PCR measurement to approximate the clinical routine sample conditions as much as possible. Additionally, we demonstrated in a previous study that consecutive sampling showed limited differences in bacterial load between the different samples [29]. Furthermore, the clinical sample size in this study was with 50 women, relatively small. In a currently ongoing study, FemCure [30], we will focus on the assessment of CT viability by V-PCR in clinical samples of a larger cohort of CT positive women. Finally, heat-inactivation of CT cultures has shown to be an effective method to obtain non-viable CT samples used for the method validation in this study. However, heat-killing does not resemble the natural clearance of CT infections including the role of the immune system, which possibly could have a different effect on bacterial membrane integrity and subsequent clearance of CT DNA and/or RNA.

In conclusion, compared with traditional culturing methods, V-PCR has the advantage of combining the high specificity and sensitivity of qPCR with the ability to discriminate viable from non-viable bacteria without the need of labour intensive cell culture methods. V-PCR was applied to asses CT viability in clinical samples for the first time and results have indicated that a substantial amount of the amplified CT DNA originated from non-viable cells. Although results might be influenced by bacterial cell death during transport, this study suggests that there is a potential overestimation of quantitative CT positivity by current routine NAAT assays.
